# P06 Waldenström macroglobulinemia – beware the label of primary Raynaud’s Phenomenon

**DOI:** 10.1093/rap/rkab068.005

**Published:** 2021-10-19

**Authors:** Shivani Gor, Rosemary Waller

**Affiliations:** Great Western Hospital, Swindon, United Kingdom

## Abstract

**Case report - Introduction:**

Waldenström’s macroglobulinemia (WM) is a rare haematological malignancy accounting for 2% of blood cancers. It is characterised by an immunoglobulin M (IgM)-associated lymphoplasmacytic lymphoma and usually follows an indolent disease course. Most patients present with nonspecific constitutional symptoms, commonly fatigue secondary to anaemia. Cryoglobulins are found in approximately 15% of WM patients but are symptomatic in less than 5% of cases. Symptoms range from Raynaud’s phenomenon to skin ulcers. We present a 56-year-old presenting with episodic blue discoloration of the ears and face, associated with cold exposure. Investigation revealed a diagnosis of WM with symptomatic cryoglobuloinaemia.

**Case report - Case description:**

A 56-year-old female was referred to the rheumatology clinic with symptoms of Raynaud’s phenomenon. Further questioning revealed that she had been having Raynaud’s symptoms for many years, however over the last 18 months she experienced blue discolouration of her face, neck and ears when exposed to the cold. The lesions were itchy but non-tender and settled promptly on re-warming. There was no skin blistering or ulceration. She had also noted a reduced exercise tolerance. She was previously a keen runner but found she was short of breath after 2 miles, particularly on an incline or flight of stairs. There was no history of weight loss, loss of appetite or night sweats.

Examination revealed skin changes consistent with resolving chilblains on the feet, with some nail dystrophy. There were a couple of splinter haemorrhages on one of the fingernails and no features of active Raynaud’s. There was no lymphadenopathy or hepatosplenomegaly and both cardiovascular and respiratory examination were normal.

Blood tests showed a normal full blood count and renal function. Further investigations went on to reveal a cryoglobulinaemia with IgM kappa paraprotein. She was then referred to the haematology team who arranged for a bone marrow biopsy. This showed a 10—15% infiltration of low-grade B-cell lymphoma. A CT chest, abdomen, pelvis reported no lymphadenopathy, a normal spleen and a thyroid goitre.

Treatment was initiated in the form of a triple therapy, dexamethasone, rituximab and cyclophosphamide. She underwent one cycle with good response and her original symptoms have now resolved. She is awaiting her second cycle of treatment.

**Case report - Discussion:**

Lymphoplasmacytic lymphoma (LPL) is a mature B-cell neoplasm composed of small B lymphocytes showing plasmacytoid or plasma-cell differentiation. WM is a form of LPL in which there is evidence of bone marrow involvement and a monoclonal IgM paraprotein in the serum. WM accounts for over 95% of cases of LPL. Patients with WM can have symptoms caused by tumour infiltration (fever, night sweats, weight loss, fatigue) and/or by monoclonal IgM (hyperviscosity, cyoglobinaemia, cold agglutinin). Cryoglobulins are immunoglobulins or immune complexes (which contain rheumatoid factor) that precipitate below 37 °C and re-dissolve on warming. Typically, cryoglobulin measurements are done on serum samples using tubes without gel separators. Temperature control of the specimen during collection, transportation and centrifugation is vital and if not done correctly, can affect diagnosis.

The term cyroglobulinaemia refers to the presence of one or more of these immunoglobulins in the serum. Cryoglobulinaemia in association with WM usually involve type I cryoglobulins or mixed immunoglobulin complexes, where monoclonal IgM behaves as an antibody against polyclonal IgG.  Patients with symptomatic cryoglobulinaemia can present in a variety of ways including rapidly progressive glomerulonephritis, polyarthralgia, cutaneous ulcers and Raynaud’s phenomenon.

In WM patients with symptomatic cryoglobulinaemia, exposure to cold leads to intravascular gelling of cryoglobulins with subsequent intravascular agglutination of red cells. This impairs the circulation to the cold exposed organs and vasoconstriction of the vessels can occur. The stagnant blood causes ischaemia and with pallor and consequently cyanosis develops.  In our patient this pathway led to Raynaud’s and facial cyanosis symptoms.

**Case report - Key learning points:**

Although the presence of cryoglobulins is frequent in WM, fewer than 5% are symptomatic. This case illustrates the importance of thorough history and investigation in patients that are referred to the Rheumatology outpatient clinic with presumed primary Raynaud’s phenomenon. Our patient had probable primary Raynaud’s phenomenon for many years, however she then had a change in symptoms, i.e., facial cyanosis, and this is what prompted further investigation for potential malignancy. The symptoms can be present for many months before the insidious nature of the underlying WM manifests and thus a high index of suspicion is necessary. Another important learning point is, if a patient presents with Raynaud’s symptoms and they do not meet the typical demographic for primary Raynaud’s disease (young onset, relatively mild disease with female preponderance) it is crucial to investigate for secondary causes.

Skin manifestations related to symptomatic cryoglobulinaemia are common, with the most frequent being purpura and ulcers. Our patient presented with intermittent cyanosis of the face that could have progressed to necrotic ulceration if a diagnostic delay had occurred. Treatment directed at the underlying cause of the symptomatic cryoglobulinaemia results in improvement or stabilisation of symptoms in the majority of patients, with disappearance of cryoglobulins in over 50% of cases.

Finally, clinicians often rely on constitutional symptoms of lethargy, weight loss and night sweats being present as triggers to investigate for a malignancy. Our patient displayed none of these red flag symptoms and even had a normal haemogloblobin level. This emphasises that awareness of this relatively rare disease entity is essential when faced with similar clinical scenarios as our patient and that cryoglobulinaemia should be on our differential list.

